# Detection of Novel Integrons in the Metagenome of Human Saliva

**DOI:** 10.1371/journal.pone.0157605

**Published:** 2016-06-15

**Authors:** Supathep Tansirichaiya, Md. Ajijur Rahman, Agata Antepowicz, Peter Mullany, Adam P. Roberts

**Affiliations:** 1 Department of Microbial Diseases, UCL Eastman Dental Institute, University College London, 256 Gray's Inn Road, London, WC1X 8LD, United Kingdom; 2 Department of Pharmacy, University of Rajshahi, Rajshahi-6205, Bangladesh; Institut National de la Recherche Agronomique, FRANCE

## Abstract

Integrons are genetic elements capable of capturing and expressing open reading frames (ORFs) embedded within gene cassettes. They are involved in the dissemination of antibiotic resistance genes (ARGs) in clinically important pathogens. Although the ARGs are common in the oral cavity the association of integrons and antibiotic resistance has not been reported there. In this work, a PCR-based approach was used to investigate the presence of integrons and associated gene cassettes in human oral metagenomic DNA obtained from both the UK and Bangladesh. We identified a diverse array of gene cassettes containing ORFs predicted to confer antimicrobial resistance and other adaptive traits. The predicted proteins include a putative streptogramin A O-acetyltransferase, a bleomycin binding protein, cof-like hydrolase, competence and motility related proteins. This is the first study detecting integron gene cassettes directly from oral metagenomic DNA samples. The predicted proteins are likely to carry out a multitude of functions; however, the function of the majority is yet unknown.

## Introduction

Integrons are commonly found in bacterial genomes, especially in most Gram-negative bacteria. They are involved in the dissemination and differential expression of genes in the bacterial population [1–3]. They contain two common features, a functional platform and an array of gene cassettes (GCs). The former or the 5’ conserved segment (5’CS) contains the integrase gene, *intI*, an *attI* recombination site and the promoter *Pc*. This platform is used for the capturing and expression of the GCs, non-replicative mobile elements which generally couple one or more open reading frames (ORFs) with the cassette-associated recombination *attC* site. The *intI* gene encodes a site-specific tyrosine recombinase, IntI which catalyses the integration and excision of the GCs. The expression of integrase genes can be upregulated by the SOS response, a bacterial stress response induced by the accumulation of single stranded DNA in a cell, such as transformation, conjugation, starvation and exposure to antibiotics such as quinolones and trimethoprim [4].

The recombination usually occurs between *attI*, located immediately adjacent to *intI*, and *attC* which is present on circular gene cassettes [5–7]. The *attC* sites contain two inverted regions of homology (L’-R’ and R”-L”), which flank the central region containing a highly variable sequence. The size of *attC* can be between 57 to 141 bp [8]. Even though the sequences of *attC* sites are not conserved, they show a palindromic organization which is essential for the formation of the correct hairpin structure, which is the recognition site of the integrase during integron GC recombination reactions [9]. The GCs normally do not have a promoter. The *Pc* promoter is usually required for the transcription of GC ORFs, therefore the first GC following *Pc* often has the higher levels of expression relative to downstream GC located ORFs [10].

Integrons have the potential to drive bacterial evolution and adaptation by differential expression of ORFs within GCs. One of the most clinically significant adaptive traits is antibiotic resistance [11]. The first integrons were identified by their association with antibiotic resistance genes (ARGs) [12]. Among hundreds of classes of integrons, class 1 integrons are the most commonly associated with multiple ARGs in clinical strains. More than 130 different GCs carried by integrons were predicted to confer resistance to a variety of classes of antibiotics such as aminoglycosides, beta-lactams, chloramphenicol, trimethoprim, and streptothricin [13].

Gene cassettes are abundant and disseminated widely in diverse environments. Different isolates of the same bacterial species can have different GC arrays [14]. The predicted protein functions of ORFs within GCs are varied and include, in addition to antibiotic resistance, virulence, and secondary metabolism, which are likely to be niche-specific [1, 2]. However, metagenomic analyses of the integron cassette gene pool from several studies revealed that vast majority of GCs were novel [15–17].

Due to the fact that, in many environments, less than 1% of the bacterial population is culturable [18], one of the approaches to investigate the GCs in the entire bacterial community is the PCR-based amplification of GCs using metagenomic DNA as a template [19]. Several studies on the diversity of GCs in different environments have been performed with this approach such as soil, seawater, marine sediment and deep sea vents [16, 17, 19, 20].

The human oral cavity is one of the most complex microbial ecosystems in the human body. More than 700 bacterial species have been detected from the oral cavity, [21, 22]. Many ARGs have been detected and discovered in the oral cavity, including tetracycline resistance genes *tet*(Q), *tet*(W), *tet*(M), *tet*(37) and *tet*(32)*;* erythromycin resistance genes, *ermB* and *mef*, and kanamycin resistance gene, *aphA-3* [23–25]. Recent genetic analysis of the oral metagenome showed that 2.8% of the predicted genes had the potential to encode proteins with antibiotic and toxin resistance [26]. However, very few studies investigating integrons in human oral cavity have been performed. There are two major reports on integrons in the human oral cavity; one describing an unusual or reverse integron, an integron with the integrase gene oriented in the same direction as a gene cassette array, in *Treponema denticola* ATCC35405 by using whole genome sequencing analysis, and the *in silico* analysis of an integron associated with *Treponema* species by using metagenomic datasets of the Human Microbiome Project [14, 27]. The presence of other integrons in other oral bacterial species remains to be determined.

Despite the oral microbiota being recognised as a potential source of ARGs and the oral environment providing conducive conditions for the transfer of ARGs between a range of species [25], no in depth studies have been carried out to detect integrons and GCs within the oral microbiota. In this study, we have investigated the presence of integrons and associated GCs in the human oral metagenomic DNA from two countries, the UK and Bangladesh using a PCR approach. Different sets of primers targeting different regions of integrons were used for PCR amplification, in which multiple GCs were identified and predicted to encode various proteins including some likely to confer antibiotic resistance.

## Materials and Methods

### Saliva sample collection and ethical approval

Saliva samples were collected from 11 and 10 healthy volunteers (both male and female with age between 21 and 65) from UK and Bangladesh, respectively. The UK samples were collected from the staff and international postgraduate students from the UCL Eastman Dental Institute and represent various ethnic and cultural backgrounds including Asian, Australian, European, African and Middle-Eastern, some of which had moved to the UK in the past few months. Therefore, the UK samples represent an international metagenome. The Bangladeshi samples were collected from the staff, undergraduate and post-graduate students of Department of Pharmacy of Rajshahi University all of which were Bangladeshi. All of the volunteers read and gave written consents before sample collection. None of the volunteers had received antibiotic treatment for 3 months before the sample collection day. Ethical approvals for the analysis of pooled saliva as part of this project were obtained from University College London (UCL) Ethics Committee (project number 5017/001) and the Institutional Animal, Medical Ethics, Biosafety and Biosecurity Committee (IAMEBBC) for Experimentations on Animal, Human, Microbes and Living Natural Sources, University of Rajshahi (project number 54/320/IAMEBBC/IBSC). Both ethics committees approved the consent procedures for the sample collection and processing. For the UK samples, 2 ml of saliva were collected in a sterile plastic tube and processed immediately. The samples from Bangladesh were collected and transported using Norgen’s Saliva DNA Collection and Preservation Device, (Norgen, Canada) following the manufacturer’s guidelines, and transported to UK for analysis. All samples were anonymised.

### Extraction of oral metagenomic DNA

The freshly collected UK saliva samples were pooled together into a sterile plastic tube in a class I microbiological safety cabinet. The pooled saliva sample was then divided into 1.5ml aliquots and centrifuged at 20238 g for 1 min. The UK oral metagenomic DNA was then extracted by using the Puregene DNA extraction kit (Qiagen, UK), following the Gram-positive bacteria and yeasts protocol with the modification in final step, which the DNA pellets were dissolved in 400μL molecular grade water at room temperature, instead of 100μL.

The Bangladeshi oral metagenomic DNA was extracted from the Norgen’s Saliva DNA storage buffer using ethanol precipitation technique according to manufacturer’s protocol. The preservative buffer of Norgen devices is designed for rapid cellular lysis and subsequent preservation of DNA from fresh saliva samples. Prior to DNA isolation, the storage devices were incubated for 1h at 50°C and mixed by inversion and gentle shaking for 10 seconds. DNA was then extracted from 500 μL of the pooled saliva in preservative buffer by taking 50 μL aliquots from 10 saliva samples.

### PCR amplification

**Fig 1:**
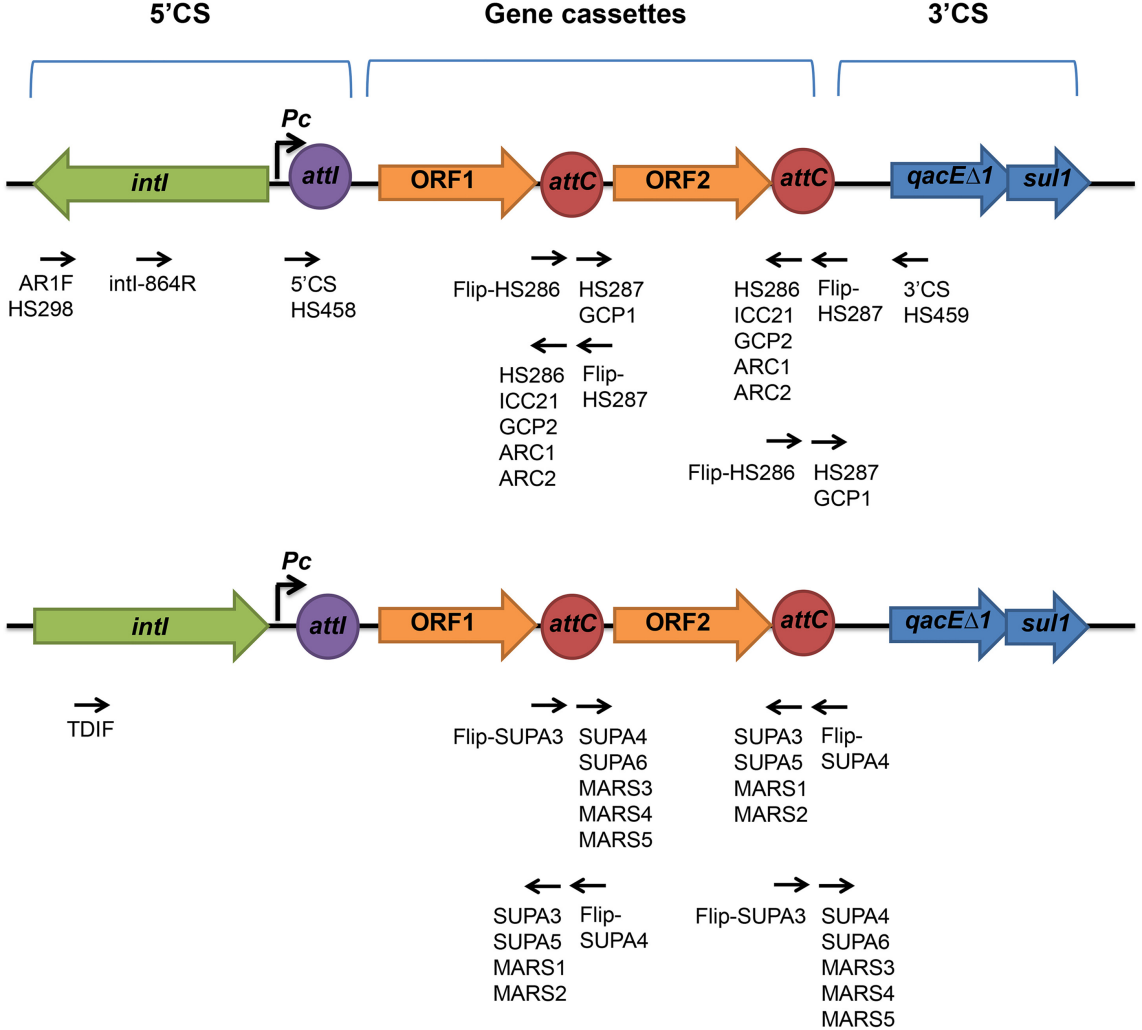
The primer binding sites of the published and newly designed primers. Primers were indicated as black arrows on A.) the class 1 integrons and B.) the unusual integron structure of *T*. *denticola*. The open arrowed boxes represent ORFs, pointing in the probable direction of transcription. The genes in 5’and 3’ conserved segment (CS), the open reading frame (ORF), the recombination site *attI* and *attC* are shown in grey, blue, green, yellow and orange respectively.

The list of primers and their sequences are shown in S1 Table and the target sites for the primers are shown in Fig 1. The typical PCR was prepared as follows; 50 μL reaction containing 15μl of 2x BioMix Red (Bioline, UK), 0.2 μM of each 10 μM primer, 50–100 ng of DNA template, and molecular grade water (Sigma, UK) up to 30 μL. The standard PCR was carried out with (i) an initial denaturation: 94°C for 5 minutes, (ii) denaturation step: 94°C for 1 minute, (iii) annealing step: 50–65°C depending on the primers for 30 seconds, (iv) elongation step: 72°C for 30 seconds to 3 minutes depending on the size of expected products, repeated step (ii)-(iv) for 35 cycles and (v) final elongation step 72°C for 10 min.

### PCR purification and gel extraction

The PCR products were subjected to electrophoresis on 1% agarose gel stained with 1:10,000 dilution of GelRed nucleic acid stain (Biotium, UK). The products were then purified by using either QIAquick PCR Purification Kit (Qiagen, UK) or QIAquick Gel Extraction Kit (Qiagen, UK), depending on the amplification results and the target amplicons, according to the manufacturer’s instructions.

### Ligation and transformation

Purified PCR products were ligated into pGEM-T Easy vector (Promega, UK). The ligation mixtures were transformed into *Escherichia coli* α-Select Silver Efficiency competent cells (Bioline, UK) by heat shock at 42°C for 40 s, and plated on Luria-Bertani (LB) agar with ampicillin (100 μg/mL) as a selective marker for the plasmids and 40 μg/ml X-Gal plus 0.4 mM IPTG for the blue-white colony screening.

### Plasmid isolation and sequencing

White colonies were subcultured in 5 mL of LB broth with ampicillin (100μg/mL) and incubated overnight. Plasmids were isolated by using QIAprep Spin Miniprep Kit (Qiagen, UK) following the manufacturer’s instructions. The presence of the insert in a plasmid was verified by a 10μl DNA digestion reaction, containing 0.5 μL EcoRI restriction enzyme (20 units/μL, New England Biolabs, UK), 1μL 10x EcoRI buffer, 100–500 ng of DNA and molecular grade water (Sigma, UK) up to 10 μL. The reactions were incubated at 37°C for 1 hour and electrophoresed on 1% agarose gel.

### Sequence analyses

DNA sequencing of inserts were performed at the Beckman Coulter Genomics (Beckman Coulter Genomics, UK) with an ABI 3730XL. M13 forward (5’ GTTTTCCCAGTCACGAC 3’) and M13 reverse (5’ GGAAACAGCTATGACCATG 3’) primers were used as the initial primers for sequencing. Additional primers were designed and used for further sequencing for the longer inserts using Primer3 (http://biotools.umassmed.edu/bioapps/primer3_www.cgi).

DNA sequences were aligned and manipulated by using BioEdit software version 7.2.0 (http://www.mbio.ncsu.edu/bioedit/bioedit.html). For the inserts which required sequencing with more than one primer, the sequences were assembled using the CAP contig function in the BioEdit program [28]. The sequences were screened for vector contamination by using VecScreen analysis tool (http://www.ncbi.nlm.nih.gov/tools/vecscreen). The primer binding sites were then identified by searching the sequences by eye. The sequences were analysed by the comparison of sequence and translated sequence using the National Centre for Biotechnology Information (NCBI) tools and databases including BlastN and BlastX [29], ORF finder and Clustal Omega.

A sequence obtained using the attC-based primers was considered a putative GCs if (i) it contains both of the primer sequences (designed from conserved nucleotides of *attC*) (ii) the sites included an integrase-like simple site at each end [10] (iii) the primer sites flank a putative ORF beginning with ATG, TTG or GTG [17]. The sequences which did not contain an ORF, but contained the *attC* site, were considered as empty GCs. The putative translated sequences were subjected to BlastX searches and matches were considered significant if the e-value was <0.001.

### Nomenclature and accession number of the gene cassettes

The gene cassettes (GCs) were named according to the source and the primers. The first two letters indicate the forward and reverse primers used for amplification. The third letter indicates the source of oral metagenomic DNA that the GCs amplified from (U for UK; B for Bangladesh), which is followed by a numerical code for the number of clone. For example, TMB1 means it is the first GC obtained from Bangladeshi samples by using the primer TDIF and MARS2.

The sequences of integron regions, which contained *intI*, Pc, *attI* and gene cassettes, were deposited in the DNA database Genbank under accession numbers from KT921469 to KT921473. The accession numbers from KT921474 to KT921509 and from KT921510 to KT921531 represented gene cassette sequences generated by the *T*. *denticola* primers from UK and Bangladeshi samples, respectively.

## Results

### Recovery and characterization of PCR products containing *intI* and the first gene cassette

Initially, we used previously published primers that had been used to successfully amplify gene cassettes from a range of environments (Fig 1, S1 and S2 Tables). Unexpectedly none of these primers produced amplicons having the structural features of a gene cassette [17] when oral metagenomic DNA isolated from the UK and Bangladesh was used as a template (see materials and methods).

**Fig 2:**
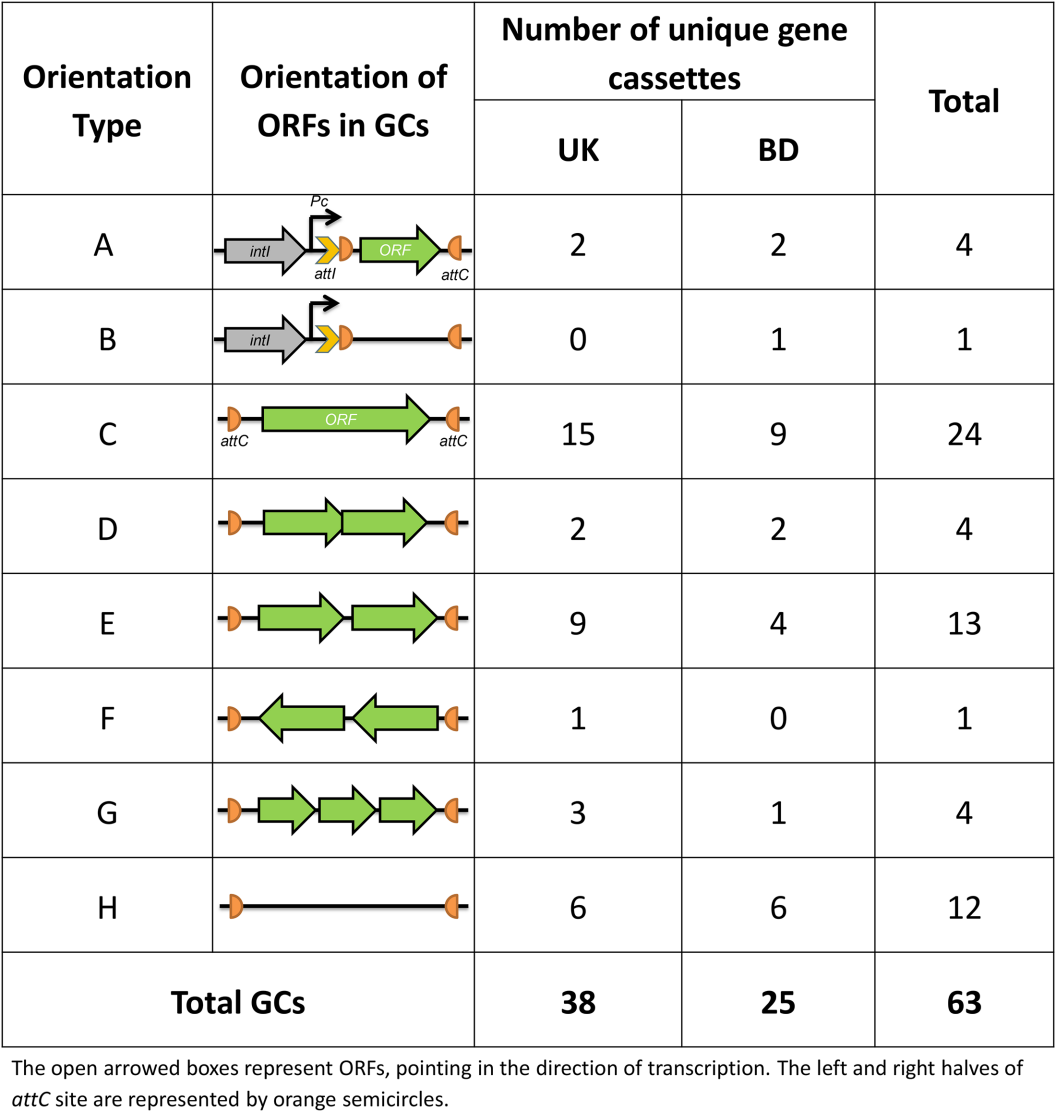
Orientation of ORFs in the GCs recovered from metagenomic DNA of saliva samples.

As *Treponema denticola* integrons are the only ones that have been described in the oral microbiota [27], new primers were designed based on this integron. The PCR were performed by using the *intI*-based primer TDIF (designed based on the conserved amino acid sequence SSQNQAL of IntI of the *Treponema denticola* integron) coupled with the *attC*-based primer MARS2. Resulting amplicons were cloned into pGEM-T Easy vector and a total of 17 clones were randomly selected from both cohorts and the inserts within the plasmids were sequenced. All of these contained the basic features of an integron. Within the amplicons, a major part of *intI* (768 bp), the full length *attI* site and a putative integron promoter, *Pc* were detected. A total of 5 different amplicons containing 5 different GCs including one empty GC with no identifiable ORF were found (Fig 2). The putative ORFs detected on the GCs had a size range of 258 to 777 bp (Table 1).

**Table 1:** Characterization of all gene cassettes detected in the saliva metagenomic DNA from UK and Bangladesh using TDIF and MARS2 primer combination to detect the first gene cassette.

						BlastN (ORF of first gene cassette)			BlastX (ORF of first gene cassette)					
Gene cassettes /clone code	Primer pair	Size of insert including the integrase gene and first cassette (bp)	Orientation type	Distance between integrase gene and ORF of first gene cassette (bp)	Accession number	Closest homologue	Percentage identity (%)	Coverage (%)	Closest homologue	ORF size (bp)	Percentage identity (%)	Coverage (%)	The presence of ribosomal binding site	Accession number of the homologous proteins (BlastX)
TMB1/8/12/15	TDIF-MARS2	1499	A	271	KT921469	No significant similarity	-	-	No significant similarity	258	-	-	Yes	-
TMB3/5/6/10/11/13/14/16	TDIF-MARS2	1757	A	198	KT921470	*Treponema putidum*	98	100	Cof-like hydrolase [*Treponema denticola*]	768	96	100	Yes	WP_016512123
TMB4	TDIF-MARS2	1244	B	-	KT921471	*Treponema pedis*	82	46	-	-	-	-	-	-
TMU3/4/11	TDIF-MARS2	1775	A	204	KT921472	*Treponema sp*. OMZ 838	77	97	Hypothetical protein [*Treponema denticola*]	777	99	100	Yes	WP_002678613.1
TMU18	TDIF-MARS2	1692	A	161	KT921473	No significant similarity	-	-	Hypothetical protein [*Treponema denticola*]	387	100	100	Yes	WP_002689012.1

Among the 17clones sequenced from both cohorts, 8 clones (TMB3/5/6/10/11/13/14/16) had a GC having an ORF (768-bp) predicted to encode a protein homologous to a cof-like hydrolase of *Treponema putidum*. Two of the first gene cassettes with an ORF of 258-bp and 387-bp present on clones TMB1/8/12/15 and TMU18, respectively had no nucleotide sequence similarity to anything in GenBank. However, at the amino acid level the 387-bp ORF on TMU18 showed 100% identity with a hypothetical protein of *T*. *denticola*. Another GC detected on clones TMU3/4/11 with an ORF of 777-bp was found to encode a hypothetical protein of *Treponema denticola* (Table 1). Finally, an empty first GC was found on clone TMB4.All but one ORF detected on the first GCs had putative ribosomal binding sites (RBS) at less than 8-bp upstream of the ORFs. In all first gene cassettes, two putative integrase binding sites (L and R; also termed as S2 and S1, respectively) were detected on the *attI* sites where the integrase binding sites S1 (R) were found to contain a plausible *attI-attC* junction (GTT). The 7 bp core site Rʹʹ (1L) of *attC* was also detected upstream of the reverse MARS2 primer having the consensus sequence RYY(/R)YAAC (S3 Table). In most cases, the stop codons of the ORFs was located at these Rʹʹ integrase binding sites of *attC* [8, 30].

### Gene Cassettes Amplified Using *attC*-based primers

A library of PCR amplicons obtained using a different set of *attC*-based primers was constructed in pGEM-T Easy vector and the inserts from 100 clones were sequenced (Table 2, Fig 1, S1 and S2 Tables). By analysing the sequences with different bioinformatics tools we have detected a total of 58 unique GCs having the features of an integron GC and flanked by the primer binding sites. The size of the cassettes ranged from 425 to 1144 bp. Of the 58 GCs, 12 had no identifiable ORFs and the remaining 46 GCs contained one or more putative ORFs giving a total of 72 different ORFs with a size range between 117 to 894 bp. As the forward and reverse primers were designed based on the consensus Lʹ (2R) and Lʹʹ (2L) core sites, respectively, we were able to locate the Rʹ (1R) core sites in all GCs with a consensus GTTRR(Y)R(Y)Y(R) after the forward primer sequence. The complementary Rʹʹ (1L) core sites with a consensus R(Y)Y(R)Y(R)YAAC were also detected upstream or as a part of the reverse *attC* primers which confirms that the putative GCs are not PCR artefacts and is consistent with the *attC* structure of a GC [31]. The majority of the Rʹ and Rʹʹ core sites (51 out of 58 GCs) exhibited 100% complementarity with each other. In the remaining seven, 6 out of 7-bp were complimentary (S4 Table).

**Table 2:** Characterization of all gene cassettes detected in the saliva metagenomic DNA from UK and Bangladesh using *attC*-based primers.

						BlastN			BlastX					
Gene cassettes/clone code	Primer pair	Cassette Size (bp)	Orientation type	Distance between attC and ORF (bp)	Accession number	Closest homologue	Percentage identity (%)	Coverage (%)	Closest homologue	ORF size (bp)	Percentage identity (%)	Coverage (%)	The presence of ribosomal binding site	Accession number of the homologous proteins (BlastX)
SSU1/2, MMU15	SUPA3-SUPA4, MARS5-MARS2	799	C	36	KT921474	No significant similarity found.	-	-	Hypothetical protein [*Rhodonellum psychrophilum*]	579	66	75	Yes	WP_026333632.1
SSU3/4/30	SUPA3-SUPA4	964	E	41	KT921475	No significant similarity found.	-	-	Glyoxalase [*Treponema pedis*]	390	97	100	Yes	WP_009105863.1
									Competence protein TfoX [*Treponema pedis*]	315	83	100	Yes	WP_024470244.1
SSU5	SUPA3-SUPA4	832	E	136	KT921476	*Treponema sp*.	88	27	Hypothetical protein [*Treponema putidum*]	243	67	100	Yes	WP_044978234.1
									Twitching motility protein PilT [*Treponema putidum*]	396	71	100	Yes	AIN93467.1
SSU6	SUPA3-SUPA4	1138	E	34	KT921477	No significant similarity found.	-	-	No significant similarity found.	459	-	-	Yes	-
									Hypothetical protein [*Treponema denticola*]	339	75	100	Yes	WP_044013590.1
SSU7	SUPA3-SUPA4	491	F	0	KT921478	*Treponema denticola*	92	40	No significant similarity found	162	-	-	No	-
									No significant similarity found	132	-	-	Yes	-
SSU8	SUPA3-SUPA4	921	C	41	KT921479	*Treponema sp*.	98	49	Hypothetical protein [*Treponema denticola*]	567	98	93.7	Yes	WP_002692239.1
SSU9/13/14/19/20/23	SUPA3-SUPA4	425	H	-	KT921480	*Treponema pedis*	78	69	No significant similarity found	-	-	-	-	-
SSU10	SUPA3-SUPA4	612	C	29	KT921481	No significant similarity found.	-	-	Hypothetical protein [*Paenibacillus assamensis*]	537	39	91	Yes	WP_028595336
SSU11	SUPA3-SUPA4	648	C	4	KT921482	No significant similarity found.	-	-	Hypothetical protein [*Bradyrhizobium sp*. STM 3809]	597	42	89.7	Yes	WP_035659994.1
SSU12	SUPA3-SUPA4	736	C	77	KT921483	No significant similarity found.	-	-	Hypothetical protein [*Treponema vincentii*]	582	99	100	Yes	WP_016518887.1
SSU15	SUPA3-SUPA4	989	G	105	KT921484	*Treponema denticola*	90	19	Hypothetical protein [*Treponema sp*.]	396	40	44.3	Yes	WP_044015417.1
									Hypothetical protein [*Treponema denticola*]	228	39	16.4	No	WP_010689034.1
									Hypothetical protein TPE_0657 [*Treponema pedis*]	147	96	100	Yes	AGT43153.1
SSU16	SUPA3-SUPA4	711	E	127	KT921485	*Treponema pedis*	90	21	Hypothetical protein [*Treponema sp*.]	363	33	41.6	Yes	WP_044015417.1
									Hypothetical protein TPE_0657 [*Treponema pedis*]	147	96	100	Yes	AGT43153.1
SSU17	SUPA3-SUPA4	787	C	30	KT921486	*Treponema denticola*	98	100	Hypothetical protein [*Treponema denticola*]	711	97	100	Yes	WP_002690335.1
SSU18	SUPA3-SUPA4	853	C	31	KT921487	No significant similarity found	-	-	Hypothetical protein (Endonuclease) [*Treponema putidum*]	765	33	99.2	Yes	WP_044978378.1
SSU21	SUPA3-SUPA4	871	D	273	KT921488	*Treponema denticola*	97	100	Toxin [*Treponema denticola*]	291	96	100	No	WP_010692226.1
									Hypothetical protein (antitoxin) [*Treponema denticola*]	258	100	100	Yes	WP_010692225.1
SSU22	SUPA3-SUPA4	1017	E	72	KT921489	No significant similarity found	-	-	Prevent-host-death family protein [*Treponema medium*]	264	94	100	Yes	WP_016523165.1
									XRE family transcriptional regulator [*Treponema vincentii*]	441	96	100	Yes	WP_006188306.1
SSU24	SUPA3-SUPA4	962	E	23	KT921490	No significant similarity found	-	-	Glyoxalase [*Treponema pedis*]	411	97	100	No	WP_024470245.1
									Competence protein TfoX [*Treponema pedis*]	222	88	55.2	No	WP_024470244.1
SSU25	SUPA5-SUPA6	789	C	31	KT921491	*Treponema putidum*	96	99	Hypothetical protein [*Treponema denticola*]	705	97	100	Yes	WP_010697531.1
SSU26	SUPA5-SUPA6	848	C	34	KT921492	*Treponema putidum*	81	93	Cof-like hydrolase [*Treponema denticola*]	768	76	100	Yes	WP_010693073.1
SSU27	SUPA5-SUPA6	833	G	155	KT921493	*Treponema denticola*	96	100	No significant similarity found	126	-	-	-	-
									Antitoxin HicB [*Treponema denticola*]	405	99	100	No	WP_002669522.1
									Toxin HicA [*Treponema denticola*]	195	97	100	Yes	WP_002669524.1
SSU28	SUPA5-SUPA6	927	E	310	KT921494	*Treponema putidum*	97	91	Multidrug transporter MatE [*Treponema putidum*]	336	96	100	Yes	WP_044979179.1
									mRNA-degrading endonuclease [*Treponema denticola*]	231	99	100	Yes	WP_010694033.1
SSU29	SUPA5-SUPA6	425	H	-	KT921495	*Treponema pedis*	78	69	-	-	-	-	-	-
MMU2	MARS5-MARS2	519	H	-	KT921496	*Treponema denticola*	81	72	-	-	-	-	-	-
MMU3	MARS5-MARS2	826	C	63	KT921497	No significant similarity found	-	-	Hypothetical protein [*Morganella morganii*]	753	32	97.13	Yes	WP_036423270.1
MMU7	MARS5-MARS2	765	C	148	KT921498	*Treponema denticola*	96	98	Hypothetical protein [*Treponema sp*.]	390	100	100	Yes	WP_002688988.1
MMU9/13	MARS5-MARS2	725	C	127	KT921499	*Treponema denticola*	90	21	Hypothetical protein *[Treponema phagedenis*]	474	53	98.8	Yes	WP_044634793.1
MMU11	MARS5-MARS2	734	H	-	KT921500	*Treponema pedis*	79	51	-	-	-	-	-	-
MMU18	MARS5-MARS2	690	C	174	KT921501	No significant similarity found	-	-	Hypothetical protein [*Treponema denticola*]	459	97	100	Yes	EGC77532.1
MMU19	MARS5-MARS2	431	H	-	KT921502	*Treponema pedis*	78	63	-	-	-	-	-	-
MMU20	MARS5-MARS2	943	C	72	KT921503	*Treponema putidum*	84	16	No significant similarity found	780	-	-	No	-
MMU24	MARS5-MARS2	1041	D	435	KT921505	*Treponema sp*. OMZ 838	97	62	Plasmid maintenance system killer protein [*Treponema sp*. OMZ 838]	279	97	100	Yes	WP_044013090.1
									Hig A family addiction module antidote protein [*Treponema socranskii*]	315	97	100	Yes	WP_016519833.1
MMU25	MARS5-MARS2	1144	G	61	KT921506	*Treponema sp*. *OMZ 838*,	79	43	No significant similarity found	462	-	-	Yes	-
									Hypothetical protein [*Treponema maltophilum*]	213	91	91	Yes	WP_016526060.1
									Hypothetical protein [*Treponema vincentii*]	351	77	100	Yes	WP_044013590.1
MMU26	MARS5-MARS2	972	C	27	KT921507	*Treponema denticola*	95	100	Carbon-nitrogen hydrolase [*Treponema denticola*]	891	97	100	Yes	WP_044901990.1
MMU27	MARS5-MARS2	896	E	38	KT921508	No significant similarity found	-	-	Hypothetical protein [*Treponema denticola*]	402	95	88	Yes	WP_002687406.1
									Hypothetical protein [*Treponema denticola*]	360	99	100	Yes	WP_002673043.1
MMU28	MARS5-MARS2	987	E	320	KT921509	*Treponema putidum*	89	76	Prevent-host-death protein [*Treponema putidum*]	282	91	100	Yes	WP_044979047.1
									Hypothetical protein (Plasmid stabilization protein) [*Treponema putidum*]	213	85	99.1	Yes	AIN94307.1
MMU23	MARS5-MARS2	634	H	-	KT921504	*Treponema pedis*	84	60	-	-	-	-	-	-
MMB1/2	MARS5-MARS2	633	H	-	KT921510	*Treponema pedis*	80	56	-	-	-	-	-	-
MMB3/9	MARS5-MARS2	846	C	369	KT921511	*Treponema sp*.	27	86	Twitching motility protein PilT [*Treponema putidum*]	414	70	89	No	AIN93467.1
MMB5/35	MARS5-MARS2	423	H	-	KT921513	*Treponema denticola*	84	94	-	-	-	-	-	-
MMB4/12/31	MARS5-MARS2	877	D	271	KT921512	*Treponema denticola*	96	100	Toxin [*Treponema denticola*]	288	95	100	No	WP_01069226.1
									Hypothetical protein [*Treponema pedis*]	258	99	100	Yes	WP_024468276.1
MMB11/13/21/25/29	MARS5-MARS2	629	C	98	KT921514	*Treponema denticola*	98	81	Membrane protein [*Treponema denticola*]	312	94	100	Yes	WP_002669840.1
MMB15	MARS5-MARS2	889	C	31	KT921516	No significant similarity found	-	-	Hypothetical protein [*Mariprofundus ferrooxydans*]	804	46	89	Yes	WP_009850619
MMB22	MARS5-MARS2	854	C	160	KT921521	*Clostridium sp*.	74	43	Vat family streptogramin A O- acetyltransferase [*Treponema denticola*]	645	99	100	Yes	WP_00267814.1
MMB14	MARS5-MARS2	862	E	86	KT921515	*Treponema putidum*	66	97	RelB/DinJ antitoxin [*Treponema putidum*]	417	97	95	Yes	WP_044978223
									Hypothetical protein [*Treponema vincentii*]	300	86	100	Yes	WP_016519648.1
MMB17	MARS5-MARS2	838	E	49	KT921517	*Treponema denticola*	99	100	Hypothetical protein [*Treponema denticola*]	396	98	100	Yes	WP_010957066.1
									Hypothetical protein [*Treponema putidum*]	117	100	100	Yes	WP_010957065.1
MMB18	MARS5-MARS2	792	C	26	KT921518	*Treponema putidum*	98	100	Hypothetical protein [*Treponema denticola*]	708	97	100	Yes	WP_010697531.1
MMB19	MARS5-MARS2	817	C	45	KT921519	*Treponema denticola*	94	100	Hypothetical protein [*Treponema denticola*]	708	96	100	Yes	WP_010697511.1
MMB20	MARS5-MARS2	531	H	-	KT921520	*Treponema pedis*	80	66	-	-	-	-	-	-
MMB23/27	MARS5-MARS2	901	D	283	KT921522	No significant similarity found	-	-	Peptidase (Antitoxin) [*Treponema denticola*]	243	63	100	Yes	WP_002667901.1
									PemK toxin [*Treponema denticola*]	330	77	100	Yes	WP_002680107.1
MMB28	MARS5-MARS2	543	C	122	KT921523	No significant similarity found	-	-	Hypothetical protein [*Clostridium drakei*]	360	62	98	Yes	WP_029160182.1
MMB30	MARS5-MARS2	808	C	25	KT921524	No significant similarity found	-	-	Hypothetical protein [*Treponema medium*]	729	92	100	Yes	WP_016670455
MMB32	MARS5-MARS2	632	H	-	KT921525	*Treponema pedis*	79	56	-	-	-	-	-	-
MMB33	MARS5-MARS2	810	E	80	KT921526	*Treponema denticola*	99	100	Hypothetical protein [*Treponema denticola*]	372	98	100	No	WP_002689013.1
									Hypothetical protein [*Treponema sp*.]	276	99	100	Yes	WP_002689021.1
MMB34	MARS5-MARS2	714	C	120	KT921527	No significant similarity found	-	-	Hypothetical protein [*Treponema medium*]	525	94	99	Yes	WP_016522334.1
MMB36	MARS5-MARS2	732	H	-	KT921528	*Treponema pedis*	79	48	-	-	-	-	-	-
MMB37	MARS5-MARS2	635	H	-	KT921529	*Treponema denticola*	82	60	-	-	-	-	-	-
MMB38	MARS5-MARS2	947	G	3	KT921530	*Treponema sp*.	97	67	No significant similarity found	201	-	-	No	-
									HigA antitoxin [*Treponema socranskii*]	282	98	100	Yes	WP_016519833.1
									Plasmid maintenance system killer protein [*Treponema medium*]	318	97	100	Yes	WP_040858928.1
MMB39	MARS5-MARS2	857	E	97	KT921531	No significant similarity found	-	-	Hypothetical protein [*Treponema medium*]	357	96	100	Yes	WP_016522532.1
									Hypothetical protein [*Treponema medium*]	330	95	100	Yes	WP_016522533.1

By analysing the arrangement of genetic features within the GCs we found that they were arranged in seven different ways (Fig 2) as defined by the direction, position and number of ORFs within the GCs. The type C arrangement accounted for the majority; found in 24 cassettes. The sequences of the clones containing two or more ORFs were examined for the presence of other putative *attC* sequences in between the ORFs, none of which were found. These observations show that the *attC*-based primers based on the *T*. *denticola* integron are able to amplify GCs from oral metagenomic DNA. From 72 putative ORFs found in all GCs, 63 of them had ribosomal binding sites located upstream of the predicted start codons. As in previous studies the GCs other than the toxin-antitoxin encoding GCs did not contain an identifiable promoter, thus are likely to be dependent on the *P*c of the cassette array for expression [19].

### Diversity of the functions of putative proteins encoded by ORFs within the GCs detected by *attC* primers

Out of 72 putative ORFs detected on 58 different GCs amplified by using *attC* primers, 66 (91.66%) of the predicted proteins had a homologue in GenBank. However, only 24 of the 66 ORFs (36.36%) were found to encode proteins with known function and the remaining 42 matched hypothetical proteins. With regards to sequence similarity of the ORFs with those in GenBank, we found that 45 of the 66 ORFs (68.0%) exhibited >90% amino acid identity. Ten putative ORFs were predicted to encode completely novel proteins (e-value <0.001).

The putative ORFs detected on the gene cassettes were predicted to encode proteins of diverse functions including antibiotic resistance, host adaptation to stress and competence (Table 2). Four different putative antibiotic resistance genes were found among the cassette ORFs. BlastX searches showed that the clone MMB22 contained an ORF that encoded a protein with 99% identity to streptogramin A O-acetyltransferase from *T*. *denticola*. The single ORF (390-bp) present in the clones SSU3, SSU4 and SSU30 of UK was predicted to encode a glyoxalase/bleomycin antibiotic binding protein. Two ORFs were detected in the clone SSU28 encoding potassium ABC transporter ATPase and multidrug transporter MatE. Proteins related to adaptation to stress include different toxin-antitoxin systems and a twitching motility protein. The clones containing the ORFs encoding toxin-antitoxin system includes SSU27, MMB23, MMB38 which encoded HicA (toxin)- HicB (antitoxin), peptidase (antitoxin)-PemK (toxin) and higA (antitoxin)-higB (toxin), respectively.

Most of the proteins encoded by the ORFs on GCs showed similarity with many proteins in the database, some of which were from *Treponema* spp. (60 out of 66) mostly from *T*. *denticola* (24 out of 60) followed by *T*. *putidum*, *T*. *medium*, *T*. *vincentii*, *T*. *pedis*, *T*. *phagedenis*, and *T*. *socranskii*. This observation supports the previous reports that *T*. *denticola*, *T vincentii and T*. *phagedenis* carry chromosomal integrons [14, 32]. However, we have also identified 27 ORFs related to other *Treponema* spp.; *T*. *putidum*, *T*. *medium*, *T*. *pedis* and *T*. *socranskii*. Only six ORFs out of 66 were predicted to encode proteins related to non-treponemes including those from *Paenibacillus sp*., *Clostridium sp*. and *Maripofundus* sp. however, the homologies of the ORFs with these species were low (<70%) at the amino acid level.

## Discussion

The PCR strategies to recover novel integron cassettes from metagenomic DNA using primers targeting the conserved sequence of *IntI* and *attC* have been successful in previous studies [15–17, 19, 33]. However, all of these metagenomic studies were carried out on non-human environmental samples. Most of the metagenomic studies involving human microbiota, were either sequence-based [34] focusing on the recovery of all genetic features or focusing on a function of interest such as antibiotic resistance [35]. No studies have been reported so far on metagenomes obtained from human saliva to detect integrons using a PCR approach. We detected mostly *Treponema* integrons and GCs from metagenomic DNA from human saliva from both Bangladeshi and UK samples, indicating that this methodology is applicable to any oral metagenomic sample.

This study provides an analysis of the diversity of integron GCs amplifiable in saliva metagenomic DNA. Using novel primer combinations based on the structural features of the reverse integron of *T*. *denticola* ATCC 35405 [27], we have uncovered a diverse array of gene cassettes including those in the first position, most of which are novel. Although the chromosomal integron of *T*. *denticola* ATCC 35405 is the only integron described from the oral bacteria (it has 45 gene cassettes in the array), *in silico* analysis of metagenomic data sets from the Human Microbiome Project (HMP) showed that two other *Treponema* species, including *T*. *vincentii* ATCC 35580 and *T*. *phagedenis* F0421, have also been found to carry integron GCs [14, 27, 32]. However, the PCR strategies used in this study, recovered novel GCs that were predicted to encode proteins related to those from genera other than *Treponema* spp.

Analyzing the proteins encoded by the GCs amplified from the oral cavity showed several interesting ORFs. GC SSU3 was predicted to encode a protein with 97% amino acid identity to the glyoxalase of *Treponema pedis* (WP_009105863.1, 100% coverage). It contains the Glo-EDI-BRP-like domain which can be found in metalloproteins including glyoxalase I, type I extradiol dioxygenases and bleomycin sequester proteins. Bleomycin is a glycopeptide antibiotic, which inhibits the peptidoglycan synthesis in bacteria, and also used as an antitumor drug which bind to DNA and generate free radicals that result in both double-strand and single-strand DNA breaks [36, 37]. Another ORF found on GC MMB22 detected in the Bangladeshi sample was predicted to encode streptogramin A O-acetyltransferase which had 77.0% nucleotide identity with *Clostridium sp*. BLN1100 and 99.0% amino acid identity with the streptogramin A O-acetyltransferase from *T*. *denticola*. Streptogramin A O-acetyltransferases mediate resistance to the streptogramin A-B combination by adding acetyl group to streptogramin, which inactivates the drugs [38].

Finally, a cof-like hydrolase gene (a member of haloacid dehalogenase superfamily) was predicted to be within a GC amplified using both GC primers and first gene cassette primers (GC SSU26 and GC TMB3). Cof-like hydrolases are a group of enzymes that inactivate halogenated aliphatic hydrocarbons by hydrolysing the carbon-halogen bonds. They are essential for detoxification of many chlorinated compounds [39, 40]. Therefore, a cof-like hydrolase in the oral cavity could play a role in detoxifying or inactivating antimicrobials or other compounds with carbon-halogen bonds that are used as antibiotics, pesticides and food preservatives such as chloramphenicol, atrazine and brominated vegetable oil, respectively.

Another function of predicted GC ORFs was related to the adaptation of bacteria to environmental stress. For example, the twitching motility PilT protein was predicted to be encoded by the ORF in GC of clone SSU5, MMB3 and MMB9. It has been shown to be involved with type IV fimbria-mediated twitching motility and protease secretion [41]. Twitching motility was also shown to play a key role in the development of biofilm from *Pseudomonas aeruginosa* [42]. As many oral bacteria can form biofilms on the surfaces in the human oral cavity, having a PilT-encoded GC could help them to develop biofilms and survive environmental stress.

As in previous metagenomic studies to detect integron GCs [16, 17, 43], ORFs predicted to encode proteins with regulatory functions such as toxin-antitoxin (TA) systems have been detected. Four different TA operons including the HicAB, HigBA, RelBE and MazF were detected on GCs in our study. TA cassettes are usually abundant in chromosomal integrons and are thought to have a role in the stability of the integron GC arrays [27, 44]. All of the detected TA cassettes are the members of type II toxin-antitoxin systems [45]. The toxins (HicA, HigA, RelE and MazF) work by cleaving mRNA, inhibiting translation and exhibit bacteriostatic activity, and the antitoxins (HicB, HIgB, RelE, MazE) can inhibit the action of toxin by protein-protein complex formation [46–49]. Among the four detected TA operons, only the HicAB TA system was previously found on the *T*. *denticola* integron. The nucleotide sequence of HicA and HicB system found on SSU27 cassette exhibited 97% and 99% nucleotide identity to the corresponding fourth gene cassette of the integron of *T*. *denticola*, containing HicA (TDE1838) and HicB (TDE1837) genes[27]. We have detected two HigBA TA systems in our GCs (MMU24 and MMB38), and this system has also been detected on the *Vibrio cholerae* super integron Several recovered GCs did not contain ORFs. This kind of ORF-less GCs was found both in the first position GC and other GC positions in the integron (clone TMB4, SSU29 and MMU2). Other noncoding cassettes have been previously found in cassette arrays comprising, for example, between 4 and 49% of *Vibrio* spp. cassette arrays [50]. They have been hypothesised to contain promoters or encode regulatory RNAs [2]. It was previously shown that a *Xanthomonas campestris* integron GC encoded trans-acting small RNA, which was capable of regulating the virulence in *Xanthomonas* [51].

This survey on the presence of integrons and associated GCs in salivary metagenomic DNA has resulted in new information regarding the putative functions and diversity of GCs which likely reflects the highly variable physicochemical and stressful environment of the human oral cavity.

## Supporting Information

S1 Table Primers used in this study. (DOCX)

S2 Table The lists of primer pairs used in this study. (DOCX)

S3 Table Complementary integrase binding site S1 (R) and R” seqauence on the first gene cassettes. (DOCX)

S4 Table Complementary of the cores sites Rʹ (1R) and Rʹʹ (1L) abutting the forward and reverse *attC* primer sequence on the gene cassette. (DOCX)
