# Supplementary material for: Detection of Novel Integrons in the Metagenome of Human Saliva
Source: PLoS One. 2016 Jun 15;11(6):e0157605. doi: 10.1371/journal.pone.0157605 (PMC4909258; doi:10.1371/journal.pone.0157605)
Supplement: S1 Table — (DOCX) [file pone.0157605.s001.docx]

**S1 Table. Primers used in this study.**

| Primer name | Primer sequence (5' → 3')^a^ | Gene target | Source or reference |
| --- | --- | --- | --- |
| Primers designed based on an unusual *Treponema denticola* integrons (Accession number: NC_002967) | | | |
| SUPA3 | CAGGTTGAAGCGGGTGTTAG | *attC* site (reverse primer) | This study |
| SUPA4 | CCGCAAATGCAGGTTAAGCG | *attC* site (forward primer) | This study |
| SUPA5 | CAGVTTGAAGCGRRYGTTAG | *attC* site (reverse primer) | This study |
| SUPA6 | CCRCAAATGYWGGTYAAGCG | *attC* site (forward primer) | This study |
| Flip-SUPA3 | CTAACACCCGCTTCAACCTG | *attC* site (forward primer) | This study |
| Flip-SUPA4 | CGCTTAACCTGCATTTGCGG | *attC* site (reverse primer) | This study |
| MARS1 | CGYAATRTCAKGTTGAAG | *attC* site (reverse primer) | This study |
| MARS 2 | GCAATGTCAGGTTGAAGC | *attC* site (reverse primer) | This study |
| MARS3 | CRCRAMYRYWGGTYAAGCG | *attC* site (forward primer) | This study |
| MARS 4 | CRCAAATGCAGGTYAAGCG | *attC* site (forward primer) | This study |
| MARS 5 | CGCAAATGCAGGTTAAGCG | *attC* site (forward primer) | This study |
| TDIF | TCAAGCCAAAATCAGGCTCT | *intI* (forward primer) | This study |
| Primers designed based on normal integrons | | | |
| HS286 | TCSGCTKGARCGAMTTGTTAGVC | *attC* site (reverse primer) | [1] |
| HS287 | GCSGCTKANCTCVRRCGTTAGSC | *attC* site (forward primer) | [2] |
| Flip-HS286 | GBCTAACAAKTCGYTCMAGCSGA | *attC* site (forward primer) | This study |
| Flip-HS287 | GSCTAACGYYBGAGNTMAGCSGC | *attC* site (reverse primer) | This study |
| GCP1 | GCSGCTKANCTCVRRCGTTRRRY | *attC* site (forward primer) | [3] |
| GCP2 | TCSGCTKGARCGAMTTGTTRRRY | *attC* site (reverse primer) | [3] |
| AR1F | GCAAGTAGCGTATGCGCTCACGC | *intI1* (forward primer) | This study |
| ARC1 | CGATTTGTTATGCCGCATC | *attC* of class 1 integron (reverse primer) | This study |
| ARC2 | GTTTGACATGAGGGGCGG | *attC* of class 1 integrons (reverse primer) | This study |
| HS458 | GTTTGATGTTATGGAGCAGCAACG | 5ʹ-conserved segment (5ʹ-CS) | [4] |
| HS459 | GCAAAAAGGCAGCAATTATGAGCC | 3ʹ-conserved segment (3ʹ-CS) | [4] |
| 5’-CS | GGCATCCAAGCAGCAAG | 5ʹ-conserved segment (5ʹ-CS) | [5] |
| 3’-CS | AAGCAGACTTGACCTGA | 3ʹ-conserved segment (3ʹ-CS) | [5] |
| *intI*-864R | YAGCAGATGNGTGGCRAAVSWRTGSCG | *intI* gene conserved region | [3] |
| HS298 | ACRTGNGTRTADATCATNGT | Conserved C-terminal sequence in *intI* | [6] |
| Primers for sequencing of the inserts in pGEM-T Easy vector | | | |
| M13F | GTAAAACGACGGCCAG | M13 forward sequencing | Universal |
| M13R | CAGGAAACAGCTATGAC | M13 reverse sequencing | Universal |

^a^ Degenerate nucleotides: D = A, G, or T; H = A, C, or T; I or N= A, C, G, or T; K = G or T; M = A or C; R = A or G; S = G or C; V = A, C, or G; Y = C or T.

**References**

1. Stokes HW, Holmes AJ, Nield BS, Holley MP, Nevalainen KM, Mabbutt BC, et al. Gene cassette PCR: sequence-independent recovery of entire genes from environmental DNA. Applied and environmental microbiology. 2001;67(11):5240-6. Epub 2001/10/27. doi: 10.1128/aem.67.11.5240-5246.2001. PubMed PMID: 11679351; PubMed Central PMCID: PMCPMC93296.

2. Elsaied H, Stokes HW, Nakamura T, Kitamura K, Fuse H, Maruyama A. Novel and diverse integron integrase genes and integron-like gene cassettes are prevalent in deep-sea hydrothermal vents. Environmental microbiology. 2007;9(9):2298-312. Epub 2007/08/10. doi: 10.1111/j.1462-2920.2007.01344.x. PubMed PMID: 17686026.

3. Elsaied H, Stokes HW, Kitamura K, Kurusu Y, Kamagata Y, Maruyama A. Marine integrons containing novel integrase genes, attachment sites, *attI*, and associated gene cassettes in polluted sediments from Suez and Tokyo Bays. The ISME journal. 2011;5(7):1162-77. Epub 2011/01/21. doi: 10.1038/ismej.2010.208. PubMed PMID: 21248857; PubMed Central PMCID: PMCPMC3146285.

4. Betteridge T, Partridge SR, Iredell JR, Stokes HW. Genetic context and structural diversity of class 1 integrons from human commensal bacteria in a hospital intensive care unit. Antimicrobial agents and chemotherapy. 2011;55(8):3939-43. Epub 2011/06/02. doi: 10.1128/aac.01831-10. PubMed PMID: 21628540; PubMed Central PMCID: PMCPMC3147655.

5. Martinez-Freijo P, Fluit AC, Schmitz FJ, Grek VS, Verhoef J, Jones ME. Class I integrons in Gram-negative isolates from different European hospitals and association with decreased susceptibility to multiple antibiotic compounds. The Journal of antimicrobial chemotherapy. 1998;42(6):689-96. Epub 1999/03/03. PubMed PMID: 10052890.

6. Nield BS, Holmes AJ, Gillings MR, Recchia GD, Mabbutt BC, Nevalainen KM, et al. Recovery of new integron classes from environmental DNA. FEMS Microbiol Lett. 2001;195(1):59-65. Epub 2001/02/13. PubMed PMID: 11166996.
