# Supplementary material for: Detection of Novel Integrons in the Metagenome of Human Saliva
Source: PLoS One. 2016 Jun 15;11(6):e0157605. doi: 10.1371/journal.pone.0157605 (PMC4909258; doi:10.1371/journal.pone.0157605)
Supplement: S2 Table — (DOCX) [file pone.0157605.s002.docx]

**S2 Table. The lists of primer pairs used in this study.**

| **Forward primer** | **Reverse primer** | **Expected amplicons** |
| --- | --- | --- |
|  |  |  |
| 5’CS | 3’CS | From 5’ conserved sequence to 3’ conserved |
| HS458 | HS459 | From 5’ conserved sequence to 3’ conserved |
| 5’CS | SUPA3 | From 5’ conserved sequence to one of *attC* |
| HS298 | HS286 | From 5’ conserved sequence to one of *attC* |
| TDIF | MARS2 | From integrase gene to *attC* |
| HS287 | HS286 | From one *attC* to another *attC* |
| ICC48 | ICC21 | From integrase gene to *attC* |
| *intI-*864R | GCP2 | From integrase gene to *attC* |
| AR1F | ARC1 | From integrase gene to *attC* |
| AR1F | ARC2 | From integrase gene to *attC* |
| SUPA4 | SUPA3 | From one *attC* to another *attC* |
| SUPA6 | SUPA5 | From one *attC* to another *attC* |
| MARS3 | MARS1 | From one *attC* to another *attC* |
| MARS4 | MARS2 | From one *attC* to another *attC* |
| MARS5 | MARS2 | From one *attC* to another *attC* |
| Flip-SUPA3 | Flip-SUPA4 | From one *attC* to another *attC* |
| Flip-HS286 | Flip-HS287 | From one *attC* to another *attC* |
| GCP1 | GCP2 | From one *attC* to another *attC* |
