# Supplementary material for: Detection of Novel Integrons in the Metagenome of Human Saliva
Source: PLoS One. 2016 Jun 15;11(6):e0157605. doi: 10.1371/journal.pone.0157605 (PMC4909258; doi:10.1371/journal.pone.0157605)
Supplement: S3 Table — (DOCX) [file pone.0157605.s003.docx]

**S3 Table. Complementary integrase binding site S1 (R) and R’’ sequence on the first gene cassettes.**

| Integrase binding site S1 (R) sequence of *attI* | Pattern of S1 (R) sequence of *attI* | Integrase binding site S2 (L) sequence of *attI* | Sequence of Rʹʹ core site of *attC* before the reverse primer | Pattern of Rʹʹ core site sequence of *attC* | Clones | Complementarity between the pattern of S1 (R) of *attI* and Rʹʹ core sites of *attC* on first GC |
| --- | --- | --- | --- | --- | --- | --- |
| GTTAGAC | GTTRRRY | GTCGAAA | GTCTAAC | RYYYAAC | TMB4 | 7/7 |
| GTTAGAT | GTTRRRY | GTCGAAG | ATCTAAC | RYYYAAC | TMB1 | 7/7 |
| GTTATGG | GTTRYRR | GTTGAAA | CCATAAC | YYRYAAC | TMU18 | 7/7 |
| GTTATGT | GTTRYRY | GTCGAAG | ACCTAAC | RYYYAAC | TMB3 | 6/7 |
| GTTATGT | GTTRYRY | GTCGAAG | ACATAAC | RYRYAAC | TMU3 | 7/7 |
