# Supplementary material for: Detection of Novel Integrons in the Metagenome of Human Saliva
Source: PLoS One. 2016 Jun 15;11(6):e0157605. doi: 10.1371/journal.pone.0157605 (PMC4909258; doi:10.1371/journal.pone.0157605)
Supplement: S4 Table — (DOCX) [file pone.0157605.s004.docx]

**S4 Table. Complementary of the cores sites Rʹ (1R) and Rʹʹ (1L) abutting the forward and reverse *attC* primer sequence on the gene cassettes.**

| Type | Sequence of Rʹ after the forward primer sequence of the *attC* on GCs | Pattern of Rʹ sequence of the GC | Sequence of Rʹʹ before the reverse primer of the *attC* on GCs GC | Pattern of Rʹʹ sequence of the *attC* on GCs GC | Clones | Complementarity between the pattern of Rʹ and Rʹʹ core sites of the *attC* on GCs |
| --- | --- | --- | --- | --- | --- | --- |
| A (37) | GTTAGAC | GTTRRRY | GTCTAAC | RYYYAAC | SSU1 | 7/7 |
|  | GTTAGAC | GTTRRRY | GTCTAAC | RYYYAAC | SSU9 | 7/7 |
|  | GTTAGAC | GTTRRRY | GTCTAAC | RYYYAAC | SSU10 | 7/7 |
|  | GTTAGAC | GTTRRRY | GTCTAAC | RYYYAAC | SSU21 | 7/7 |
|  | GTTAGAC | GTTRRRY | GTCTAAC | RYYYAAC | SSU29 | 7/7 |
|  | GTTAGAC | GTTRRRY | GTCTAAC | RYYYAAC | MMU2 | 7/7 |
|  | GTTAGAC | GTTRRRY | GTCTAAC | RYYYAAC | MMU19 | 7/7 |
|  | GTTAGAC | GTTRRRY | GTCTAAC | RYYYAAC | MMU23 | 7/7 |
|  | GTTAGAC | GTTRRRY | GTCTAAC | RYYYAAC | MMU24 | 7/7 |
|  | GTTAGAC | GTTRRRY | GTCTAAC | RYYYAAC | MMB2 | 7/7 |
|  | GTTAGAC | GTTRRRY | GTCTAAC | RYYYAAC | MMB4 | 7/7 |
|  | GTTAGAC | GTTRRRY | GTCTAAC | RYYYAAC | MMB5 | 7/7 |
|  | GTTAGAC | GTTRRRY | GTCTAAC | RYYYAAC | MMB19 | 7/7 |
|  | GTTAGAC | GTTRRRY | GTCTAAC | RYYYAAC | MMB20 | 7/7 |
|  | GTTAGAC | GTTRRRY | GTCTAAC | RYYYAAC | MMB32 | 7/7 |
|  | GTTAGAC | GTTRRRY | ATCTAAC | RYYYAAC | MMB34 | 7/7 |
|  | GTTAGAC | GTTRRRY | GTCTAAC | RYYYAAC | MMB36 | 7/7 |
|  | GTTAGAC | GTTRRRY | GTCTAAC | RYYYAAC | MMB37 | 7/7 |
|  | GTTAGAC | GTTRRRY | GTCTAAC | RYYYAAC | MMB38 | 7/7 |
|  | GTTAGAT | GTTRRRY | ATCTAAC | RYYYAAC | SSU15 | 7/7 |
|  | GTTAGAT | GTTRRRY | ATCTAAC | RYYYAAC | SSU16 | 7/7 |
|  | GTTAGAT | GTTRRRY | ATCTAAC | RYYYAAC | SSU17 | 7/7 |
|  | GTTAGAT | GTTRRRY | ATCTAAC | RYYYAAC | SSU18 | 7/7 |
|  | GTTAGAT | GTTRRRY | ATCTAAC | RYYYAAC | MMU9 | 7/7 |
|  | GTTAGAT | GTTRRRY | ATCTAAC | RYYYAAC | MMU28 | 7/7 |
|  | GTTAGAT | GTTRRRY | ATCTAAC | RYYYAAC | MMB15 | 7/7 |
|  | GTTAGAT | GTTRRRY | ATCTAAC | RYYYAAC | MMB17 | 7/7 |
|  | GTTAGAT | GTTRRRY | ATCTAAC | RYYYAAC | MMB39 | 7/7 |
|  | GTTAGAT | GTTRRRY | ATCTAAC | RYYYAAC | SSU12 | 7/7 |
|  | GTTAGGC | GTTRRRY | GCCTAAC | RYYYAAC | SSU7 | 7/7 |
|  | GTTAGGC | GTTRRRY | GCCTAAC | RYYYAAC | MMB18 | 7/7 |
|  | GTTAGGT | GTTRRRY | GCCTAAC | RYYYAAC | SSU25 | 7/7 |
|  | GTTAGGT | GTTRRRY | ACCTAAC | RYYYAAC | MMU18 | 7/7 |
|  | GTTAGGT | GTTRRRY | ACCTAAC | RYYYAAC | MMU20 | 7/7 |
|  | GTTAGGT | GTTRRRY | ACCTAAC | RYYYAAC | MMB22 | 7/7 |
|  | GTTGAAC | GTTRRRY | ATCTAAC | RYYYAAC | SSU5 | 7/7 |
|  | GTTGAAC | GTTRRRY | ACCTAAC | RYYYAAC | MMU7 | 7/7 |
| B (10) | GTTATAC | GTTRYRY | GCCTAAC | RYYYAAC | SSU8 | 6/7 |
|  | GTTATAC | GTTRYRY | GTCTAAC | RYYYAAC | SSU26 | 6/7 |
|  | GTTATAC | GTTRYRY | ATCTAAC | RYYYAAC | MMB14 | 6/7 |
|  | GTTATAC | GTTRYRY | GCATAAC | RYRYAAC | MMB28 | 7/7 |
|  | GTTATGT | GTTRYRY | ACCTAAC | RYYYAAC | SSU6 | 6/7 |
|  | GTTATGT | GTTRYRY | ACCTAAC | RYYYAAC | SSU28 |  |
|  | GTTATGT | GTTRYRY | ACATAAC | RYRYAAC | MMU11 | 7/7 |
|  | GTTATGT | GTTRYRY | ACCTAAC | RYYYAAC | MMU25 | 6/7 |
|  | GTTATGT | GTTRYRY | ACATAAC | RYRYAAC | MMB11 | 7/7 |
|  | GTTATGT | GTTRYRY | ACATAAC | RYRYAAC | MMB23 | 7/7 |
| C (5) | GTTAAGA | GTTRRRR | TCTTAAC | YYYYAAC | SSU3 | 7/7 |
|  | GTTAGAA | GTTRRRR | GTCTAAC | RYYYAAC | SSU11 | 6/7 |
|  | GTTAGAA | GTTRRRR | GTCTAAC | RYYYAAC | SSU22 | 6/7 |
|  | GTTAGGA | GTTRRRR | TCTTAAC | YYYYAAC | SSU24 | 7/7 |
|  | GTTAGGA | GTTRRRR | TCCTAAC | YYYYAAC | MMU27 | 7/7 |
| D (2) | GTTAGCA | GTTRRYR | TGCTAAC | YRYYAAC | MMU3 | 7/7 |
|  | GTTAGCA | GTTRRYR | TGCTAAC | YRYYAAC | MMB33 | 7/7 |
| E (2) | GTTAGCT | GTTRRYY | AGCTAAC | RRYYAAC | MMB30 | 7/7 |
|  | GTTAGTT | GTTRRYY | ACCTAAC | RRYYAAC | MMU26 | 7/7 |
| F (2) | ATTAGAC | ATTRRRY | ATCTAAC | RYYYAAC | SSU27 | 7/7 |
|  | ATTGAAC | ATTRRRY | ATCTAAC | RYYYAAC | MMB3 | 7/7 |
